# Neuron Type-Dependent Synaptic Activity in the Spinal Dorsal Horn of Opioid-Induced Hyperalgesia Mouse Model

**DOI:** 10.3389/fnsyn.2021.748929

**Published:** 2021-11-18

**Authors:** Austin Kearns, Jazmine Jayasi, Xin Liu, Jigong Wang, Yuqiang Shi, Jin Mo Chung, Jun-Ho La, Shao-Jun Tang, Chilman Bae

**Affiliations:** ^1^School of Electrical, Computer, and Biomedical Engineering, Southern Illinois University, Carbondale, IL, United States; ^2^Department of Neuroscience, Cell Biology, and Anatomy, University of Texas Medical Branch, Galveston, TX, United States

**Keywords:** opioid-induced hyperalgesia, spinal cord dorsal horn, neurokinin 1 receptor, GABAergic interneurons, central sensitization, morphine, pain, neuronal circuit polarization

## Abstract

Opioids are widely used for pain relief; however, chronic opioid use causes a paradoxical state of enhanced pain sensitivity, termed “Opioid-induced hyperalgesia (OIH).” Despite the clinical importance of OIH, the detailed mechanism by which it enhances pain sensitivity remains unclear. In this study, we tested whether repeated morphine induces a neuronal circuit polarization in the mouse spinal dorsal horn (SDH). Transgenic mice expressing GFP to neurokinin 1 receptor-expressing neurons (sNK1Rn) and GABAergic interneurons (sGABAn) that received morphine [20 mg/kg, once daily for four consecutive days (i.p.)] developed mechanical hypersensitivity. Repeated morphine altered synaptic strengths in the SDH as a specific cell-type but not in a gender-dependent manner. In sNK1Rn and non-tonic firing neurons, repeated morphine treatment significantly increased frequency of spontaneous excitatory postsynaptic current (sEPSC) and evoked EPSC (eEPSC). In addition, repeated morphine treatment significantly decreased evoked inhibitory postsynaptic current (eIPSC) in sNK1Rn. Conversely, in sGABAn and tonic firing neurons, repeated morphine treatment significantly decreased sEPSC frequency and eEPSC, but had no change of eIPSC in sGABAn. Interestingly, repeated morphine treatment significantly decreased neuronal rheobase of sNK1Rn but had no effect on sGABAn. These findings suggest that spinal neuronal circuit polarization maybe the mechanism of OIH and identify a potential therapeutic mechanism to prevent or treat opioid-induced pain.

## Introduction

Chronic pain is a significant health problem. Globally, 1 in 5 adults suffer from pain, and 1 in 10 adults are diagnosed with chronic pain each year (Goldberg and McGee, 2011). Paradoxically, patients who are repeatedly treated with opioids are routinely diagnosed with enhanced acute and/or chronic pain, an exacerbated pain condition known as opioid-induced hyperalgesia (OIH; Angst and Clark, 2006; Chu et al., 2006). OIH is clinically prevalent and patients who receive repeated opioid treatment experience significant hyperalgesia (Cohen et al., 2008; Chen et al., 2009; Hay et al., 2009). However, the underlying mechanism of OIH remains to be elucidated.

Recently, synaptic at excitatory and inhibitory synapses have been characterized as a prime mechanism of chronic pain (Luo et al., 2014). In neuropathic pain conditions, synaptic plasticity in the spinal dorsal horn (SDH) is present in the long-term potentiation (LTP) of spinothalamic tract projection plasticity neurons (Ikeda et al., 2009) and long-term depression (LTD) of GABAergic neurons (GABAn; Bittar et al., 2017). Our recent studies revealed differential synaptic plasticity between spinothalamic tract projection neurons and GABAn in SDH of spinal nerve ligation mouse model (Kim et al., 2015; Bittar et al., 2017). Recent studies revealed that ablation of spinal neurokinin-1 receptor neuron (NK1Rn) prevents the development of hyperalgesia (Mantyh et al., 1997; Nichols et al., 1999), and OIH (Vera-Portocarrero et al., 2007), and spinal administration of an NK1R antagonist reverses OIH (King et al., 2005). In addition, synaptic response of spinal NK1Rn to afferent inputs increases with disinhibition (Torsney and MacDermott, 2006).

Due to the critical role of spinal NK1Rn and GABAn in chronic pain including OIH, we hypothesized that repeated morphine use may alter neuron type-dependent synaptic strengths in the SDH, thus leading to OIH. To test this hypothesis, we performed mechanical behavior test and *ex vivo* electrophysiological recording on SDH neurons. We found that in the OIH mouse model, excitatory synaptic strength increased in excitatory neurons but decreased in inhibitory neurons, while inhibitory synaptic strength and neuronal rheobase were depressed in only excitatory neurons. These findings suggest that neuronal type-dependent central sensitization may be a mechanism of OIH.

## Materials and Methods

### Animals

**FIGURE 1:**
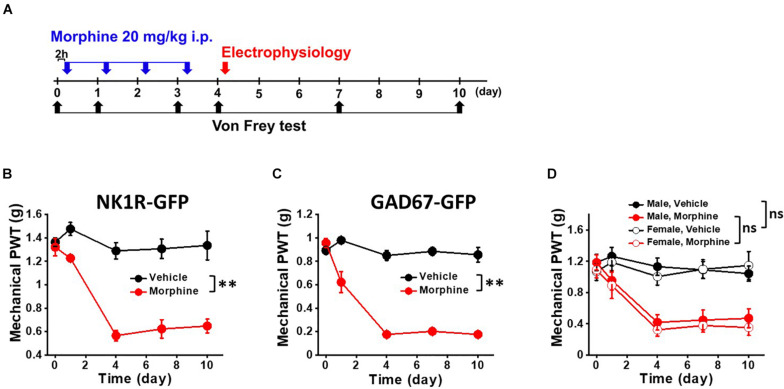
Repeated morphine treatment induced mechanical hypersensitivity in NK1R-GFP and GAD67-GFP transgenic mice. **(A)** Temporal diagram of drug administration and experiments. Morphine (blue arrow, 20 mg/kg) were administrated by intraperitoneal injection. At day 4, the mice were euthanized for electrophysiological recordings (red arrow). Pain behavioral test was performed 2 h post-morphine injection. Paw withdrawal threshold (PWT) was measured by von Frey tests (black arrow) using **(B)** NK1R-GFP mice for sNK1Rn (*N* = 6 for each group) and **(C)** GAD67-GFP mice for sGABAn (*N* = 6 for each group). **(D)** No gender effect on the repeated morphine induced mechanical hypersensitivity (Male,Vehicle (*N* = 3); Male, Morphine (*N* = 3); Female, Vehicle (*N* = 3); Female, Morphine (*N* = 3). ns: not significant, ^∗∗^*p* < 0.01 vs. vehicle by Sidak’s multiple comparison test following multilevel analysis.

All mice were maintained in Association for Assessment and Accreditation of Laboratory Animal Care International-accredited UTMB animal facility. The mice were housed in a plastic cage with standard bedding and free access to food and water on a 12/12-h light/dark cycle. Six to 8-week-old transgenic mice tagged with GFP to neurokinin 1 receptor-expressing neurons (sNK1Rn) (NK1R-GFP) (Green et al., 2019) and GABAergic interneurons (sGABAn) (FVB-Tg(GAD67-GFP) 45704Swn/J, Jackson Laboratory) in the SDH were used for behavior tests and electrophysiological recordings. For the morphine-treated groups, morphine was administered (i.p.) at a dose of 20 mg/kg by a single injection each day for four consecutive days (Figure 1A, blue arrow).

### Behavioral Testing

Mechanical nociceptive hypersensitivity in mice was measured as previously described (Callahan et al., 2008). The hind paw between the third and fourth toe in a resting state was stimulated with calibrated von Frey filaments (Stoelting, Wood Dale, IL, United States), and paw withdrawal threshold (PWT) was determined using the Dixon up and down paradigm. For thermal nociceptive sensitivity testing, paw withdrawal latency to a 52°C stimulus was measured using the hot plate test as previously described (Espejo and Mir, 1993). The latency to hind paw licking and/or shaking or jumping were determined. All tests were conducted 2 h prior to drug administration in order to avoid the antinociceptive effect of morphine. The experimenter was blind to the treatments received by individual animals (Figure 1A, black arrow). Given that sex is an important factor in pain studies and analgesia, we measured PWT in both male and female mice (Melchior et al., 2016; Roeckel et al., 2017).

### Spinal Cord Slice Preparation and Electrophysiological Recording of Dorsal Horn Neurons

Spinal cord slices were taken from OIH mouse model 1 day after the last morphine injection (Figure 1A, red arrow) and prepared as previously described (Bae et al., 2018). Briefly, the spinal cord was sliced transversely at a thickness of 350 μm using a vibratome (Leica VT1200S, Buffalo Grove, IL, United States) in cold (∼4°C) NMDG (N-methyl-D-glucamine) solution (in mM: 93 NMDG, 2.5 KCl, 1.2 NaH_2_PO_4_, 30 NaHCO_3_, 20 HEPES, 25 glucose, 5 sodium ascorbate, 2 thiourea, 3 sodium pyruvate, 10 MgSO_4_ and 0.5 CaCl_2_, pH 7.4), saturated with 95% O_2_ and 5% CO_2_. Whole-cell patch clamp recordings were made on random or GFP fluorescently identified neurons in lamina II in artificial cerebrospinal fluid (ACSF in mM: 124 NaCl, 2.5 KCl, 1.2 NaH_2_PO_4_, 24 NaHCO_3_, 5 HEPES, 12.5 glucose, 2 MgSO_4_, and 2 CaCl_2_, pH 7.4) using Multiclamp 700B amplifier, DigiDATA, and pClamp software (version 10.6 Molecular Device, Sunnyvale, CA, United States) at a 10 kHz sampling rate and a 2 kHz filtering rate. The patch-pipettes (4 – 8 MΩ) were filled with internal solution (in mM: 120 K-gluconate, 10 KCl, 2 Mg-ATP, 0.5 Na-GTP, 0.5 EGTA, 20 HEPES, and 10 phosphocreatine, pH 7.3).

Spinal dorsal horn neurons were identified by their action potential (AP) firing patterns upon depolarizing current injections (Lee et al., 2020) or using transgenic mice. After making whole-cell recording configuration, step currents (10 pA step, 300 ms duration, and 5-s intervals) were injected through the patch electrode to determine rheobase and AP firing patterns. We recorded the whole cell membrane capacitance, membrane resistance, access resistance, and resting membrane potential (Table 1). The spontaneous excitatory postsynaptic currents (sEPSC) were recorded for 60 s at −65 mV in ACSF (Bae et al., 2018; Lee et al., 2020). To minimize the contamination of IPSC by outward EPSC through ionotropic glutamate receptors, we recorded the IPSC at the reported reversal potential (0 mV) of EPSC through those receptors. EPSCs and IPSCs were evoked by focal electrical stimulation in the vicinity of recorded neurons with a metal bipolar electrode (MicroProbes, Gaithersburg, MD, United States). Test pulses were given for 0.5 ms at 5-s intervals and stimulation intensities ranging from 20 to 200 μA (20 μA step). Monosynaptic evoked EPSCs (eEPSC) and IPSC (eIPSC) were determined based on three criteria: constant short latency, a smooth waveform with a single peak (without jitter), and consistent responses without failure to repeated stimuli (Kim et al., 2015; Bittar et al., 2017). All recordings showing polysynaptic responses were disregarded.

**TABLE 1:** Comparison of the principal passive electrophysiological properties of vehicle and morphine treatments in sNK1Rn and sGABAn.

	**sNK1Rn**		**sGABAn**	
	**Vehicle**	**Morphine**	**Vehicle**	**Morphine**
Membrane capacitance (pF)	30.1 ± 1.5	29.8 ± 2.6	28.9 ± 1.4	27.2 ± 1.3
Membrane resistance (MΩ)	376.7 ± 35.0	394.7 ± 47.6	420.2 ± 25.3	446.4 ± 30.7
Access resistance (MΩ)	15.0 ± 1.6	17.3 ± 1.7	14.5 ± 0.8	14.3 ± 0.9
Resting membrane potential (mV)	−64.8 ± 1.8	−64.5 ± 1.7	−61.9 ± 1.1	−60.7 ± 1.3
Number of mice (N), cells (n)	N:5, n:27	N:4, n:24	N:7, n:65	N:8,n:48

*Values of membrane capacitance, membrane resistance, access resistance, and resting membrane potential for vehicle and morphine treatments are reported as mean ± SEM and were statistically compared by Mann–Whitney *t* test.*

### Statistical Analysis

All data were expressed as the mean ± standard error of the mean (SEM) with n, the number of cells and N, the number of mice. For electrophysiology data, all neurons from each individual mice were averaged and considered as a single data point. Means of vehicle and morphine-treated sEPSC frequency and amplitude were compared by Welch’s *t* test. The behavioral and eEPSC/eIPSC data were analyzed with Sidak’s multiple comparison test following multilevel analysis. Rheobase data were analyzed using Welch’s *t* test and ordinary one-way ANOVA with Tukey’s multiple comparisons test. Results were considered statistically significant when *p* < 0.05. We used Hedges’ g as a measure of effect size. Hedges’ g is Cohen’s d multiplied by a correction factor and takes each sample size into consideration.

## Results

### Repeated Morphine Treatment Induced Mechanical Hypersensitivity

For OIH mouse model, we modeled the systemic administration of morphine in patients by i.p. injection (Cai et al., 2016). We first tested whether repeated morphine treatment induced mechanical hypersensitivity in our transgenic mice for allowing identification of sNK1Rn and sGABAn. After the fourth daily injection of morphine (Figure 1A, blue arrows), OIH was successfully established in both strains (Figures 1B,C). PWTs were significantly reduced by repeated morphine in the mice for sNKR1n (Figure 1B) (*F*_(1,5.67)_ = 61.85, *p* < 0.001, *N* = 6 for each group) and for sGABAn (Figure 1C) (*F*_(1,10)_ = 106.55, *p* < 0.001, *N* = 6 for each group) in comparison to vehicle. Behavioral tests were performed on male and female from both strains and showed no significant difference (Figure 1D) [Morphine: *F*_(1,10)_ = 0.36, *p* = 0.561, Male (*N* = 6), Female (*N* = 6)] [Vehicle: (*F*_(1, 10)_ = 0.07, *p* = 0.800, Male (*N* = 6), Female (*N* = 6)]. Thus, all *ex vivo* electrophysiological recordings were performed on any gender.

### Repeated Morphine Treatment Increased Excitatory Synaptic Strength in Non-tonic Firing Neurons in the Spinal Dorsal Horn

**FIGURE 2:**
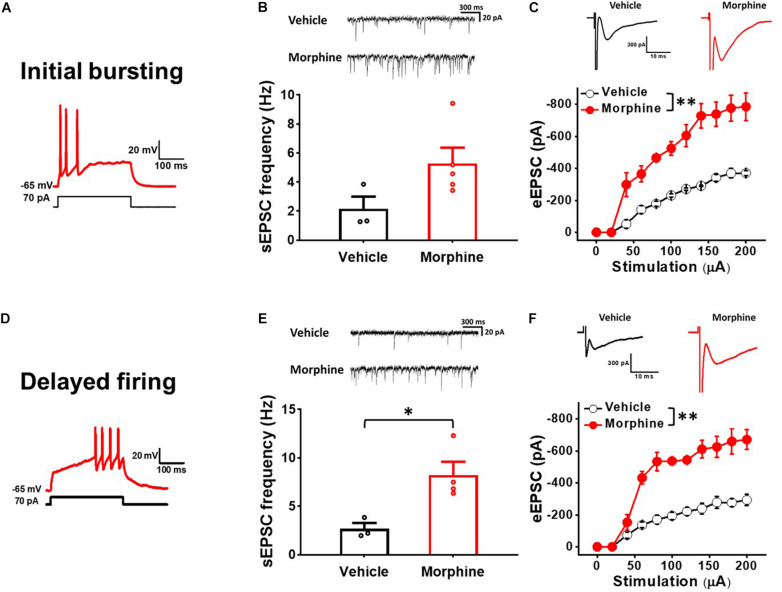
Repeated morphine treatment increased excitatory synaptic strength in non-tonic firing (Initial bursting and Delayed) neurons in the SDH. **(A)** Representative trace of initial bursting neurons. **(B)** (top) Representative traces of spontaneous excitatory postsynaptic current (sEPSC) and (bottom) sEPSC frequency in initial bursting neurons (effect size: 1.47, *p* = 0.063; Vehicle: *N* = 3, *n* = 16; Morphine: *N* = 5, *n* = 36). **(C)** (top) Representative traces of eEPSC in initial bursting neurons and (bottom) stimulus-responsive curve of eEPSC (*F*_(1,29.13)_ = 211.78, *p* < 0.001; Vehicle: *N* = 3, *n* = 13; Morphine: *N* = 3, *n* = 8). ^∗∗^*p* < 0.01 vs. vehicle by Sidak’s multiple comparison test following multilevel analysis. **(D)** Representative trace of delayed firing neurons. **(E)** (top) Representative traces of sEPSC and (bottom) sEPSC frequency in delayed firing neurons (effect size: 2.50, p = 0.020; Vehicle: *N* = 3, *n* = 7; Morphine: *N* = 4, *n* = 11). ^∗^*p* < 0.05 by Welch’s *t* test. **(F)** (top) Representative traces of eEPSC in delayed firing neurons and (bottom) stimulus-responsive curve of eEPSC (*F*_(1,5.84)_ = 82.81, *p* < 0.001). ^∗∗^*p* < 0.01 vs. vehicle by Sidak’s multiple comparison test following multilevel analysis (Vehicle: *N* = 3, *n* = 9; Morphine: *N* = 3, *n* = 7).

To determine whether repeated morphine alters synaptic strengths as a cell-type manner, we performed electrophysiological recordings on non-tonic firing neurons (presumably excitatory neurons) in the laminae II of the SDH (Lee et al., 2020). In initial bursting neurons (Figure 2A), the frequency of sEPSC was increased, but not significantly, by repeated morphine when compared to vehicle (Figure 2B) [effect size: 1.47, *p* = 0.063; Vehicle: 2.15 ± 0.85 Hz (*N* = 3, *n* = 16); Morphine: 5.29 ± 1.08 Hz (*N* = 5, *n* = 36)]. However, eEPSC in initial bursting neurons was significantly increased by morphine when compared to vehicle (Figure 2C) [*F*_(1,29.13)_ = 211.78, *p* < 0.001; Vehicle (*N* = 3, *n* = 13); Morphine (*N* = 3, *n* = 8)]. The stimulus-response curve was left shifted by repeated morphine. In delayed firing neurons (Figure 2D), the frequency of sEPSC was significantly increased by repeated morphine when compared to vehicle (Figure 2E) [effect size: 2.50, *p* = 0.020; Vehicle: 2.68 ± 0.59 Hz (*N* = 3, *n* = 7); Morphine: 8.24 ± 1.57 Hz (*N* = 4, *n* = 11)]. eEPSC in delayed firing neurons was also significantly increased by morphine when compared to vehicle (Figure 2F) [*F*_(1,5.84)_ = 82.81, *p* < 0.001; Vehicle (*N* = 3, *n* = 9); Morphine (*N* = 3, *n* = 7)].

### Repeated Morphine Treatment Increased Excitatory Synaptic Strength in sNK1Rn

**FIGURE 3:**
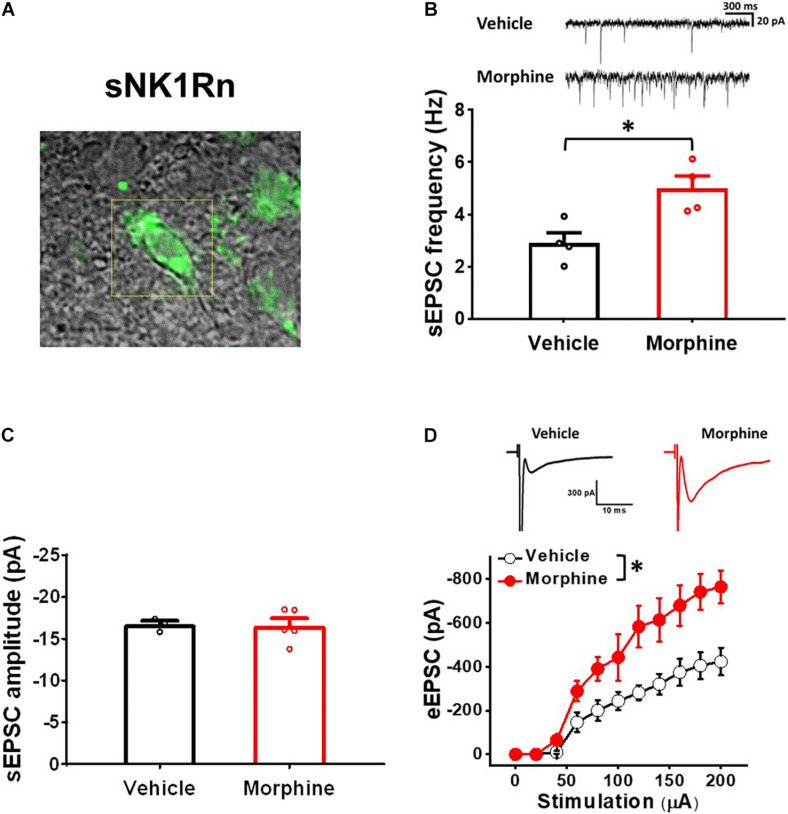
Repeated morphine treatment increased excitatory synaptic strength in sNK1Rn. **(A)** Fluorescent identification of sNK1Rn. **(B)** (top) Representative traces of sEPSC and (bottom) sEPSC frequency (effect size: 2.38, *p* = 0.016; Vehicle: *N* = 4, *n* = 27; Morphine: *N* = 4, *n* = 35). ^∗^*p* < 0.05 by Welch’s *t* test **(C)** sEPSC amplitude (effect size: 0.09, *p* = 0.891; Vehicle: *N* = 3, *n* = 24; Morphine: *N* = 5, *n* = 41). **(D)** (top) Representative traces of eEPSC and (bottom) stimulus-response curve of eEPSC [*F*_(1,5.56)_ = 9.37, *p* = 0.024; Vehicle (*N* = 4, *n* = 24), Morphine (*N* = 4, *n* = 26)]. ^∗^*p* < 0.05 vs. vehicle by Sidak’s multiple comparison test following multilevel analysis.

To confirm whether repeated morphine alters synaptic strengths as neuronal type-specific manner, we performed electrophysiological recordings on fluorescently identified sNK1Rn (Figure 3). sEPSC frequency in sNK1Rn was significantly increased by repeated morphine when compared to vehicle (Figure 3B) [effect size: 2.38, *p* = 0.016; Vehicle: 2.90 ± 0.39 Hz (*N* = 4, *n* = 27); Morphine: 4.99 ± 0.48 Hz (*N* = 4, *n* = 35)] but showed no significant difference in sEPSC amplitude (Figure 3C) [effect size:0.09, *p* = 0.891; Vehicle: −16.72 ± 0.46 pA (*N* = 3, *n* = 24); Morphine: −16.58 ± 0.88 pA (*N* = 5, *n* = 41)]. eEPSC in sNK1Rn was also significantly increased by morphine when compared to vehicle (Figure 3D) [*F*_(1,5.56)_ = 9.37, *p* = 0.024; Vehicle (*N* = 4, *n* = 24); Morphine (*N* = 4, *n* = 26)]. The stimulus-response curve was left shifted by repeated morphine. Together, results from non-tonic firing neurons and sNK1Rn suggest that repeated morphine increases excitatory synaptic strength to excitatory neurons in SDH.

### Repeated Morphine Treatment Decreased Excitatory Synaptic Strength in Tonic Firing Neurons in Spinal Dorsal Horn

**FIGURE 4:**
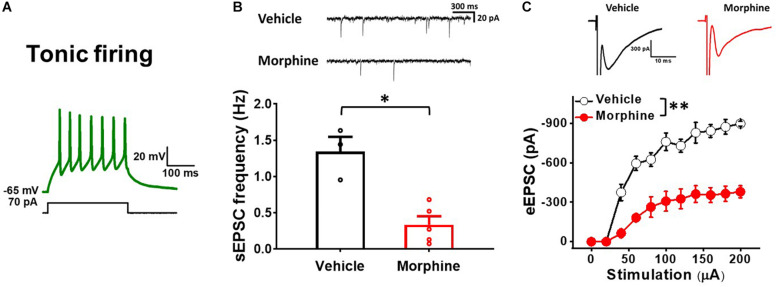
Repeated morphine treatment decreases excitatory synaptic strength in tonic firing neurons in SDH. **(A)** Representative trace of tonic firing neurons. **(B)** (top) Representative traces of sEPSC and (bottom) sEPSC frequency (effect size:–3.44, *p* = 0.018; Vehicle: *N* = 3, *n* = 12; Morphine: *N* = 5, *n* = 16). ^∗^*p* < 0.05 by Welch’s *t* test. **(C)** (top) Representative traces of eEPSC and (bottom) Stimulus-responsive curve of eEPSC [*F*_(1,5.92)_ = 25.56, *p* = 0.002; Vehicle (*N* = 4, *n* = 9); Morphine (*N* = 3, *n* = 7)]. ^∗∗^*p* < 0.01 vs. vehicle by Sidak’s multiple comparison test following multilevel analysis.

To ensure that repeated morphine alters synaptic strengths as neuron type-manner, we repeated the above experiments using tonic firing neurons (presumably inhibitory neurons) in the laminae II of the SDH (Figure 4) (Lee et al., 2020). The frequency of sEPSC in tonic firing neurons was significantly decreased by repeated morphine when compared to vehicle (Figure 4B) [effect size:−3.44, *p* = 0.018; Vehicle: 1.34 ± 0.20 Hz (*N* = 3, *n* = 12); Morphine: 0.34 ± 0.12 Hz (*N* = 5, *n* = 16)]. eEPSC in tonic firing neurons was also significantly decreased by morphine when compared to vehicle (Figure 4C) [*F*_(1,5.92)_ = 25.56, *p* = 0.002; Vehicle (*N* = 4, *n* = 9); Morphine (*N* = 3, *n* = 7)]. The stimulus-response curve was right shifted by repeated morphine.

### Repeated Morphine Treatment Decreased Excitatory Synaptic Strength in GABAergic Interneurons

**FIGURE 5:**
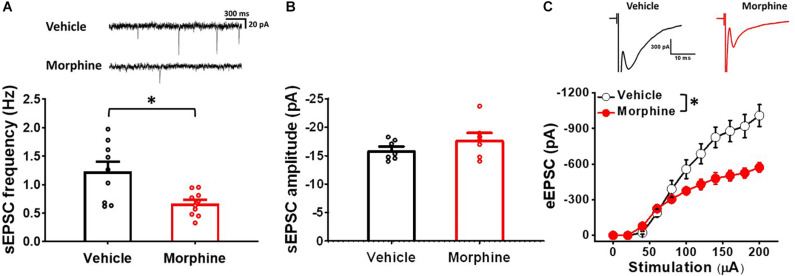
Repeated morphine treatment decreased excitatory synaptic strength in sGABAn. **(A)** (top) Representative traces of sEPSC and (bottom) sEPSC frequency (effect size:–1.46, *p* = 0.012; Vehicle: *N* = 9, *n* = 143; Morphine: *N* = 10, *n* = 123). ^∗^*p* < 0.05 by Welch’s *t* test. **(B)** sEPSC amplitude (effect size:–0.71, *p* = 0.214; Vehicle: *N* = 7, *n* = 104; Morphine: *N* = 7, *n* = 102). **(C)** (top) Representative traces of eEPSC and (bottom) stimulus-response curve of eEPSC (Figure 5C) [*F*_(1,17.47)_ = 4.96, *p* = 0.039; Vehicle (*N* = 9, *n* = 32); Morphine (*N* = 10, *n* = 87)]. ^∗^*p* < 0.05 vs. vehicle by Sidak’s multiple comparison test following multilevel analysis.

To confirm whether repeated morphine alters synaptic strengths in inhibitory neurons in the SDH, we performed electrophysiological recordings on fluorescently identified sGABAn (Figure 5). sEPSC frequency in sGABAn was significantly decreased by repeated morphine when compared to vehicle (Figure 5A) [effect size:−1.46, *p* = 0.012; Vehicle: 1.2 ± 0.2 Hz (*N* = 9, *n* = 143); Morphine: 0.7 ± 0.1 Hz (*N* = 10, *n* = 123)] but showed no significant difference in sEPSC amplitude (Figure 5B) [effect size:−0.71, *p* = 0.214; Vehicle: −15.98 ± 0.64 pA (*N* = 7, *n* = 104); Morphine: −17.81 ± 1.20 pA (*N* = 7, *n* = 102)]. eEPSC in sGABAn was also significantly decreased by morphine when compared to vehicle (Figure 5C) [*F*_(1,17.47)_ = 4.96, *p* = 0.039; Vehicle (*N* = 9, *n* = 32); Morphine (*N* = 10, *n* = 87)]. The stimulus-response curve was right shifted by repeated morphine. Together, results from tonic firing neurons and sGABAn suggest that repeated morphine decreases excitatory synaptic strength to inhibitory neurons in SDH.

### Gender Did Not Affect Repeated Morphine-Induced Changes of Synaptic Strength in Spinal Dorsal Horn

**FIGURE 6:**
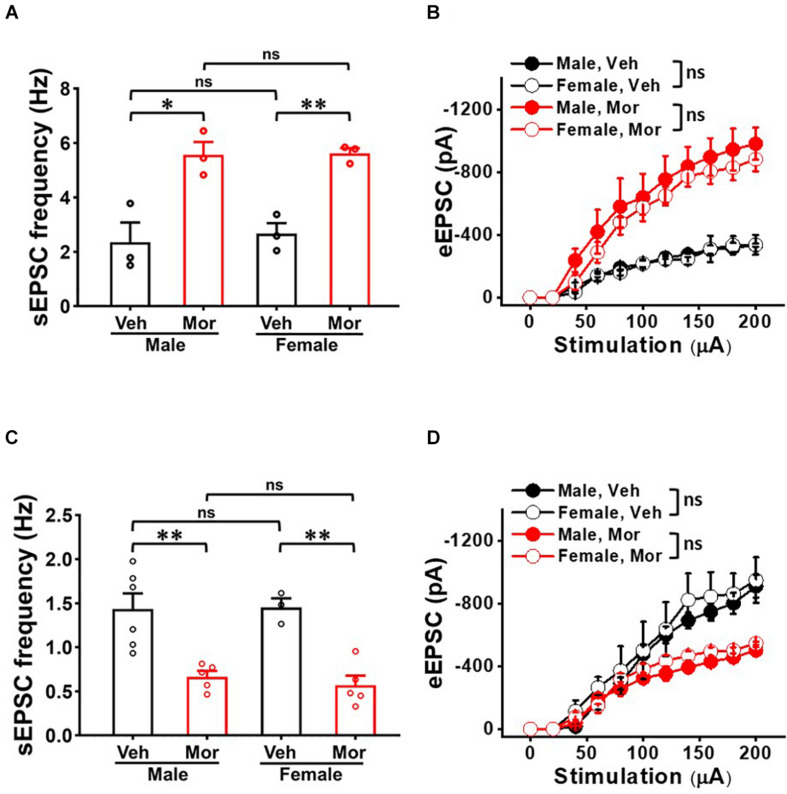
No gender effect on the repeated morphine-induced synaptic strength in SDH. **(A)** sEPSC frequency [effect size: 0.31, *p* = 0.728; Veh: Male (*N* = 3, *n* = 20) vs. Female (*N* = 3, *n* = 9) and effect size: 0.08, *p* = 0.930; Mor: Male (*N* = 3, *n* = 25) vs. Female (*N* = 3, *n* = 29)] and **(B)** eEPSC (Veh: Male (*N* = 3, *n* = 15) vs. Female (*N* = 3, *n* = 7); Mor: Male (*N* = 3, *n* = 8) vs. Female (*N* = 3, *n* = 13) in non-tonic firing neurons. **(C)** sEPSC frequency (Veh: Male (*N* = 6, *n* = 97) vs. Female (*N* = 3, *n* = 46); effect size: 0.05, *p* = 0.929 and Mor: Male (*N* = 7, *n* = 66) vs. Female (*N* = 3, *n* = 53); effect size: –0.02, *p* = 0.874) and **(D)** eEPSC (Veh: Male (*N* = 3, *n* = 19) vs. Female (*N* = 3, *n* = 13), *F*_(1, 6.09)_ = 0.41, *p* = 0.544. Mor: Male (*N* = 3, *n* = 19) vs. Female (*N* = 3, *n* = 13), *F*_(1,6.09)_ = 0.41, *p* = 0.544) in sGABAn. ^∗^*p* < 0.05 or ^∗∗^*p* < 0.01; Welch’s *t* test. ns: not significant. Sidak’s multiple comparison test following multilevel analysis. Veh: Vehicle, Mor: Morphine.

We examined whether neuron-type dependent synaptic strength changes in SDH is gender dependent (Figure 6). Even though morphine significantly affected sEPSC frequency and eEPSC in each gender, there was no significant difference between both genders in either vehicle or morphine treated group. In non-tonic firing neurons, vehicle treated mice had sEPSC frequency of 2.36 ± 0.72 Hz in male mice (*N* = 3, *n* = 20) and 2.67 ± 0.38 Hz in female mice (*N* = 3, *n* = 9) (effect size: 0.31, *p* = 0.728) (Figure 6A). Morphine treated mice had sEPSC frequency of 5.57 ± 0.47 Hz in male mice (*N* = 3, *n* = 25) and 5.62 ± 0.19 Hz in female mice (*N* = 3, *n* = 29) (effect size: 0.08, *p* = 0.930). eEPSC also showed no gender difference in vehicle group and morphine treated group (Figure 6B). In sGABAn, vehicle treated mice had sEPSC frequency of 1.43 ± 0.43 Hz in male mice (*N* = 6, *n* = 97) and 1.45 ± 0.17 Hz in female mice (*N* = 3, *n* = 46) (effect size: 0.05, *p* = 0.929). Morphine treated mice had sEPSC frequency of 0.67 ± 0.14 Hz in male mice (*N* = 5, *n* = 50) and 0.57 ± 0.14 Hz in female mice (*N* = 5, *n* = 71) (effect size:−0.67 *p* = 0.465) (Figure 6C). In addition, eEPSC showed no gender difference in vehicle group and morphine treated group (Figure 6D) (Vehicle: Male (*N* = 3, *n* = 19) vs. Female (*N* = 3, *n* = 13), *F*_(1,6.09)_ = 0.41, *p* = 0.544; Morphine: Male (*N* = 3, *n* = 23) vs. Female (*N* = 3, *n* = 22), *F*_(1,9.01)_ = 0.95, *p* = 0.355).

### Repeated Morphine Treatment Decreased Inhibitory Synaptic Strength in sNK1Rn

**FIGURE 7:**
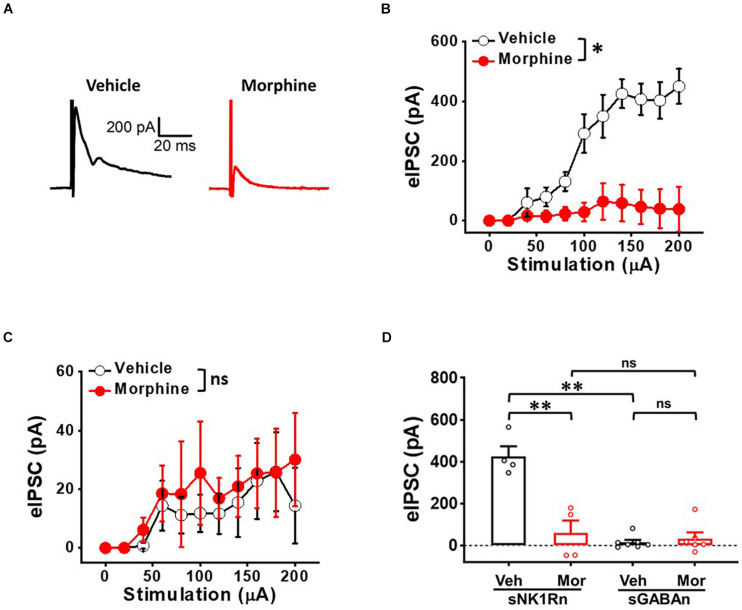
Repeated morphine treatment decreases inhibitory synaptic strength in sNK1Rn. **(A)** Representative traces of evoked inhibitory postsynaptic current (eIPSC) from sNK1Rn. Stimulus-response curve of eIPSC from panel **(B)** sNK1Rn (Vehicle: *N* = 4, *n* = 9; Morphine: *N* = 4, *n* = 19) and **(C)** sGABAn (Vehicle: *N* = 7, *n* = 48; Morphine: *N* = 6, *n* = 26). ns: not significant, ^∗^*p* < 0.05 vs. vehicle by Sidak’s multiple comparison test following multilevel analysis. **(D)** eIPSC (at 140 μA) of sNK1Rn and sGABAn from panels **(B,C)**, respectively. Effect size: –3.34, *p* = 0.0037 for sNK1Rn: Vehicle vs. Morphine. Effect size: 0.37, *p* = 0.5718 for sGABAn: Vehicle vs. Morphine. Effect size: –6.73, *p* = 0.002 between Veh:sNK1Rn and Veh:sGABAn. Effect size: –0.27, *p* = 0.734 between Mor:sNK1Rn and Mor:sGABAn. ^∗∗^*p* < 0.01; Welch’s *t* test.

To determine whether repeated morphine alters inhibitory synaptic strengths, we measured eIPSC on sNK1Rn and sGABAn (Figure 7). eIPSC in sNK1Rn was significantly reduced by morphine compared to vehicle (Figures 7A,B) [*F*_(1,3.23)_ = 19.84, *p* = 0.018; Vehicle (*N* = 4, *n* = 9); Morphine (*N* = 4, *n* = 19)] and showed a right shift in the stimulus-response curve. However, eIPSC in sGABAn showed no significant difference by morphine (Figure 7C) [*F*_(1,11.32)_ = 0.10, *p* = 0.757; Vehicle (*N* = 7, *n* = 48); Morphine (*N* = 6, *n* = 26)]. Interestingly, in vehicle groups, eIPSC at 140 μA stimulation was significantly larger in sNK1Rn in comparison to sGABAn (Figure 7D) [effect size: −6.73, *p* = 0.002; sNK1Rn (425.68 ± 48.08 pA, *N* = 4, *n* = 9); sGABAn (15.48 ± 11.67 pA, *N* = 7, *n* = 48)].

### Repeated Morphine Treatment Decreased Rheobase of sNK1Rn

**FIGURE 8:**
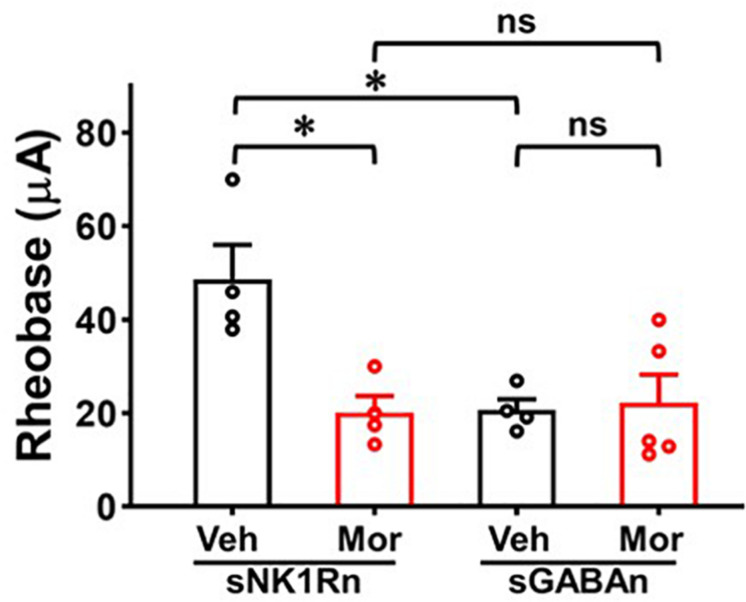
Rheobase of sNK1Rn and sGABAn. Repeated morphine treatment decreased sNK1Rn rheobase (Effect size: –2.48, *p* = 0.022; Vehicle: *N* = 4, *n* = 21, Morphine: *N* = 4, *n* = 17) but had no significant effect on sGABAn rheobase (Effect size: 0.15, *p* = 0.812; Vehicle: *N* = 4, *n* = 45, Morphine: *N* = 5, *n* = 28) ^∗^*p* < 0.05 vs. vehicle by Welch’s *t* test. ns: not significant. In vehicle groups, rheobase of sGABAn was significantly smaller in comparison to sNK1Rn (Effect size: –2.55, *p* = 0.026). ^∗^*p* < 0.05 vs. vehicle of sNK1Rn by ordinary one-way ANOVA with Tukey’s multiple comparisons test.

To determine whether the altered synaptic strengths can change neuronal excitability, we measured rheobase of sNK1Rn and sGABAn (Figure 8). Rheobase of sNK1Rn was significantly reduced by morphine compared to vehicle [effect size: −2.48, *p* = 0.022; Vehicle: 48.68 ± 7.30 μA (*N* = 4, *n* = 21); Morphine: 20.21 ± 3.54 μA (*N* = 4, *n* = 17)]. However, rheobase of sGABAn showed no significant difference by morphine [effect size: 0.15, *p* = 0.812; Vehicle: 20.69 ± 2.28 μA (*N* = 4, *n* = 45); Morphine: 22.29 ± 5.98 μA (*N* = 5, *n* = 28)]. Interestingly, in vehicle groups, rheobase of sGABAn was significantly smaller in comparison to sNK1Rn (effect size: −2.55, *p* = 0.026). These results suggest that repeated morphine preferentially sensitizes sNK1Rn excitability. Throughout the experiments we recorded the passive electrophysiological properties (cell membrane resistance and capacitance, access resistance, and resting membrane potential) and found that there were no difference in neurons from different groups (Table 1).

## Discussion

In this study, we determined whether repeated morphine treatment changes synaptic strength in the SDH. An OIH mouse model was developed through repeated morphine injection and confirmed using mechanical hypersensitivity testing (Figure 1). We performed *ex vivo* whole-cell recordings on the SDH neurons identified by their firing patterns and fluorescently identified excitatory and inhibitory neurons (sNK1Rn and sGABAn). We found that changes in synaptic strength were dependent on neuron type in the OIH mouse model. Results obtained using sNK1Rn and sGABAn were congruous with the results obtained using identification by neuronal firing patterns.

The laminae II in the SDH is a major terminal region for primary afferent fibers that are important for transmitting information related to pain (Todd, 2010). It has been proven that the SDH contains neurons that selectively respond to noxious stimuli (Grudt and Perl, 2002). However, at present, we have a limited understanding about their functional relationship featuring interconnections and morphological types of neurons from the SDH region (Todd, 2017). The Laminae II neurons have been broadly accepted into morphological classification of four main types: islet, vertical, radial and central cells (Grudt and Perl, 2002; Heinke et al., 2004; Yasaka et al., 2007). Islet cells are inhibitory, while radial and vertical cells are excitatory. Nevertheless, certain morphological types have been found in each group (Yasaka et al., 2010). Previous investigations have reported electrophysiological heterogeneity in the laminae II. Rodent SDH neurons were classified according to their depolarization-induced AP firing patterns (Grudt and Perl, 2002; Lu and Perl, 2003) and identified their cell types based on monosynaptic evoked EPSCs and eIPSCs responses, which is in accordance with our findings. Moreover, Yasaka et al. grouped neurons based on their discharge patterns and reported that 82% (18 of 22) of non-tonic neurons were excitatory neurons (Yasaka et al., 2010). In this OIH study, based on AP firing pattern results from electrophysiological recordings, 90% (35 of 39 neurons) of sNK1Rn were non-tonic firing neurons [49% initial bursting (17 of 35) and 51% of delayed firing (18 of 35)] and 88% (52 of 59 neurons) of sGABAn were tonic firing neurons. Our OIH model confirmed that sNK1Rn and sGABAn were in accord with our non-tonic and tonic firing neurons results based on their firing patterns. Interestingly, it has been suggested that noxious stimulation leads to the production of phosphorylated ERK in excitatory neurons, resulting in Kv4.2 phosphorylation and changes increased excitability patterns from delayed to tonic firing in the lamina II (Yasaka et al., 2010). Likely, it is possible that morphine may alter firing patterns. Thus, we examined the firing patterns of sNK1Rn and sGABAn treated with vehicle or morphine. We found that sNK1Rn had 50% initial bursting (9 of 18) and 50% delayed firing (9 of 18) in vehicle group and 47% initial bursting (8 of 17) and 53% delayed firing (9 of 17) in morphine group. 96% of sGABAn showed tonic firing in vehicle group and 83% in morphine group, indicating that morphine did not alter the firing patterns.

Opioid-induced hyperalgesia has been suggested to develop differently for various types of pain (Chu et al., 2008). It is important to note that only mechanical, not thermal (data not shown), hypersensitivity was induced after multiple morphine injections in this experiment. In HIV-associated pain, repeated morphine injections in the gp120 mouse pain model induced mechanical hypersensitivity that was present for 18 days (Shi et al., 2019). Other studies also have reported that repeated morphine treatments induced mechanical hypersensitivity but showed no effect on thermal hypersensitivity in mouse model (Roeckel et al., 2017; Fisher et al., 2019; Sasaki et al., 2021). In addition, an opioid related study in healthy human volunteers failed to induce hypersensitivity to noxious heat following the use of remifentanil, whereas mechanical hyperalgesia was progressively enlarged (Angst et al., 2003; Hood et al., 2003). Our results are in line with these previous studies suggesting that the strength of synapses involved in mechanical sensory processing may be more affected by repeated exposure to morphine. However, it has been noted that heat hypersensitivity occurred after 8 days of repeated opioid administration in a study by Mao et al. (1994). Moreover, hyperalgesia associated with withdrawal of opioid has been shown to induce thermal hypersensitivity in rodents (Cabanero et al., 2013).

Spontaneous and evoked EPSC have been used to measure synaptic activity changes. sEPSC frequency is indicative of spontaneous synaptic vesicle transmission (Taylor et al., 1995; Rosenmund and Stevens, 1996; Grabner and Moser, 2018) and of synaptic status in pain conditions including spinal nerve ligation (Gao et al., 2009), painful diabetic neuropathy (Chen et al., 2011), central and peripheral inflammatory pain from TNF-α (Zhang et al., 2011), and chemotherapy-induced peripheral neuropathy (Sisignano et al., 2016). Similarly, eEPSC is a measure of synaptic strength that correspond with the number of presynaptic vesicles and/or post-synaptic receptors for excitatory neurotransmitters (Atasoy et al., 2008). In our previous studies, changes of eEPSC were used as a marker of LTP and LTD in neuropathic pain models (Kim et al., 2015; Bittar et al., 2017). In neuropathic pain conditions, eEPSC was increased in randomly patched neurons in lamina II of the SDH (Chen et al., 2011) and excitatory spinothalamic tract neurons in the SDH (Bittar et al., 2017) but was decreased in GABAergic neurons (Leitner et al., 2013). Our results are in line with these findings. sEPSC frequency and eEPSC increased in sNK1Rn (Figure 3) and decreased in sGABAn (Figure 5) by repeated morphine indicating that changes of neuron type-dependent synaptic strength correlates with hypersensitivity in the OIH mouse model.

Evoked IPSC is a measure of inhibitory synaptic strength in which stimulus-evoked binding of inhibitory neurotransmitters opens chloride channels, hyperpolarizing the cell and decreasing the likelihood of AP propagation. eIPSC was decreased in studies of various neuropathic conditions. eIPSC was decreased in randomly patched lamina II neurons in spared nerve injury and chronic constriction injury models (Moore et al., 2002). eIPSC was also decreased in excitatory neurons located in the lamina II of SDH in the partial sciatic nerve ligation model (Imlach et al., 2016). These findings indicate that decreased inhibition of excitatory neurons can be a mechanism of neuropathic pain. In addition, the synaptic response of sNK1Rn to afferent inputs increased when inhibition was suppressed (Torsney and MacDermott, 2006). In rats, excitatory neurons were more disinhibited than inhibitory neurons in SDH (Stikova et al., 2019). In accordance with these findings, our results showed that repeated morphine decreased eIPSC preferentially in sNK1Rn (Figures 7A,B). Although pharmacological inhibition was not implemented in this study, sNK1Rn had strong excitatory inputs but significant loss of inhibitory inputs (Figure 7B) causing a right-shift in the eIPSC dose-response curve by repeated morphine when recorded at the reversal potential (0 mV), indicating that eIPSC was well isolated. This finding suggests that augmenting synaptic sensitization in excitatory neurons and disinhibition of inhibitory neurons may be an underlying mechanism of OIH.

Rheobase is an indicator of neuronal excitability which represents the threshold current for eliciting neural AP discharge (Li et al., 2009). In neuropathic pain, rheobase was decreased in injured DRG neurons (Kim et al., 1998) and in spared nerve injury model SDH neurons treated with TNF-alpha (Li et al., 2009). In our OIH mouse model, decreased sNK1Rn rheobase (Figure 8) indicates increased neural excitability. These findings are in accord with the results of our sEPSC, eEPSC, and eIPSC experiments, indicating the increase in sNK1Rn excitability may be due to the synaptic strength changes. After repeated morphine treatment, sGABAn rheobase showed no significant change but appeared to trend toward depression. Nevertheless, a decrease in sNK1Rn rheobase relative to sGABAn rheobase strongly indicates a net increase in excitability of spinal nociceptive circuits after repeated morphine treatment.

It has been reported that neuron type-dependent synaptic changes are involved in pain. Studies investigating neuropathic pain (Chen et al., 2011; Bittar et al., 2017), inflammatory pain (Park et al., 2011; Zhang et al., 2011), and chemotherapy-induced pain (Sisignano et al., 2016) found that sEPSC frequency and/or eEPSC amplitude were increased in excitatory or randomly patched neurons (Chen et al., 2011; Zhang et al., 2011). Specifically, GABAn and spinothalamic track neurons synaptic plasticity in mouse SDH has been observed in neuropathic pain (Bittar et al., 2017) which is known as an indicator of central sensitization (Kim et al., 2015; Bittar et al., 2017), a phenomenon in which increased membrane excitability and decreased inhibition sensitize nociceptive circuits and contribute to the development of hyperalgesia (Woolf, 2011). Nearly all chronic pain conditions show aspects of central sensitization (Harte et al., 2018), and central sensitization of nociceptive pathways is thought to be an important mechanism in a plethora of pain conditions. Important mechanisms of action of OIH are thought to involve changes in spinal plasticity (Chu et al., 2008; Lee et al., 2011), the central glutaminergic system (Chu et al., 2008), spinal dynorphins (Lee et al., 2011), mu opioid receptor signaling pathways (Roeckel et al., 2016), descending pain facilitation (Vera-Portocarrero et al., 2007; Chu et al., 2008; Roeckel et al., 2016), and decreased neurotransmitter reuptake with enhanced nociceptive response (Lee et al., 2011). Previous studies determined whether NK1Rn is involved in OIH. However, the detailed mechanism of how this NK1Rn is involved in OIH is unknown. Our findings on sNK1Rn and sGABAn indicate that central sensitization caused by neuron type-dependent synaptic activity changes is the underlying mechanism behind hyperalgesia in OIH, and a common and convergent mechanism across many chronic pain conditions.

Interestingly, studies have reported that astrocytes (Sasaki et al., 2021) and reactive oxygen species (ROS) (Shi et al., 2021) in the SDH are critical for OIH. It would be very interesting to test how they may influence neuron type-dependent synaptic activities. For future studies, it is important to also consider studying whether neuron type-dependent synaptic activities are regulated in withdrawal-induced hyperalgesia mouse model.

**FIGURE 9:**
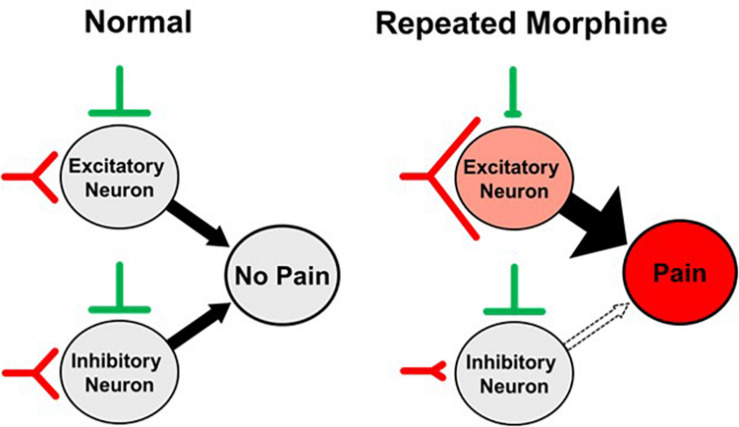
Proposed synaptic mechanism of opioid-induced hyperalgesia (OIH). Repeated morphine causes hyperalgesia (pain) by increasing excitatory input and decreasing inhibitory input to excitatory neurons but disinhibition of inhibitory neurons in the spinal dorsal horn (SDH), leading to differential effects on cell excitability.

In conclusion, we investigated the effects of repeated morphine use on mechanical sensitivity and synaptic strength in SDH neurons of OIH mouse model. Our findings suggest that the proposed mechanism of OIH (Figure 9) is caused by altered synaptic strengths in the SDH as a cell-type manner, increase of excitatory input and decrease of inhibitory input to excitatory neurons but disinhibition to inhibitory neurons, thus leading to differential effects on cell excitability.

## Data Availability Statement

The raw data supporting the conclusions of this article will be made available by the authors, without undue reservation.

## Ethics Statement

The animal study was reviewed and approved by the University of Texas Medical Branch.

## Author Contributions

S-JT, J-HL, JC, and CB: conceptualization. AK, XL, JW, YS, and CB: methodology. AK, JJ, J-HL, S-JT, and CB: writing. All authors contributed to the article and approved the submitted version.

## Conflict of Interest

The authors declare that the research was conducted in the absence of any commercial or financial relationships that could be construed as a potential conflict of interest.

## Publisher’s Note

All claims expressed in this article are solely those of the authors and do not necessarily represent those of their affiliated organizations, or those of the publisher, the editors and the reviewers. Any product that may be evaluated in this article, or claim that may be made by its manufacturer, is not guaranteed or endorsed by the publisher.
